# School-based psychosocial interventions for refugee and conflict-affected adolescents in Middle Eastern countries: a systematic review and meta-analysis

**DOI:** 10.1093/pubmed/fdag057

**Published:** 2026-07-29

**Authors:** Amani N Alansari, Amani Salim, Marwa Messaoud, Mohammed A Mahmoud, Hanan Youssif

**Affiliations:** Department of Pediatric Surgery, Hamad Medical Corporation, Doha 3050, Qatar; Faculty of Midwifery, Bethlehem University, Bethlehem, Palestine; Pediatric Surgery Department, Fattouma Bourguiba University Hospital, Monastir 5000, Tunisia; University of Monastir, Faculty of Medicine of Monastir, Monastir 5000, Tunisia; Faculty of Medicine, Al-Azhar University, Gaza, Palestine; Pediatric Surgery Department, Benghazi Medical Center, Benghazi, Libya

**Keywords:** school-based interventions, psychosocial support, adolescents, refugees, Middle East, systematic review

## Abstract

**Background:**

This study evaluated the effectiveness of school-based psychosocial interventions for conflict-affected adolescents in the Middle Eastern countries.

**Methods:**

We searched PubMed, Scopus, and Web of Science from inception to August 2025 for studies evaluating school-based interventions targeting mental health outcomes in refugee adolescents in Middle East. Eligible studies included randomized controlled trials, cohort studies, and pre–post intervention designs. Random-effects meta-analyses were conducted for single-arm (pre–post) and double-arm (intervention versus control) comparisons, using R programming for statistical analysis.

**Results:**

Nine studies involving predominantly Syrian, Palestinian, and Afghan refugee adolescents met the inclusion criteria. Single-arm analyses demonstrated significant reductions in post-traumatic stress disorder (PTSD) symptoms [mean raw score change (MRAW) = −7.50, 95% confidence interval (CI): −10.53 to −4.46], anxiety (MRAW = −9.33, 95% CI: −16.43 to −2.23), and emotional-behavioral difficulties (MRAW = −2.21, 95% CI: −3.20 to −1.22). Double-arm analyses showed significant effects for PTSD/stress [standardized mean difference (SMD) = −0.63, 95% CI: −1.26 to −0.001] and emotional-behavioral difficulties (SMD = −3.47, 95% CI: −6.55 to −0.40), but not for depression or anxiety when compared with controls.

**Conclusions:**

School-based psychosocial interventions demonstrate effectiveness in reducing trauma-related symptoms and improving emotional regulation among conflict-affected adolescents in Middle Eastern contexts, although substantial heterogeneity warrants cautious interpretation. While findings support their potential integration into humanitarian education programming, conclusions should be considered in light of variability in study design and populations.

## Introduction

The Middle East has experienced prolonged armed conflicts and humanitarian crises over the past three decades, resulting in unprecedented displacement of populations and profound mental health challenges for millions of adolescents.^1^ Countries including Syria, Iraq, Yemen, Palestine, and Lebanon have witnessed devastating conflicts that have disrupted education systems, fractured family structures, and exposed young people to traumatic events, including violence, loss, and forced displacement.^2^^,^^3^ As of 2024, the region hosts the highest concentration of refugee and internally displaced populations globally, with adolescents comprising a substantial proportion of these vulnerable groups.^4^^,^^5^

Adolescence represents a critical developmental period characterized by rapid neurobiological, psychological, and social changes.^6^ For conflict-affected youth, this sensitive period is further complicated by exposure to potentially traumatic events, chronic stress, and disrupted developmental trajectories.^7^ Research consistently demonstrates elevated rates of post-traumatic stress disorder (PTSD), depression, anxiety, and behavioral problems among refugee and conflict-affected adolescents compared to their non-affected peers.^8–10^ Without appropriate intervention, these mental health challenges can have cascading effects on educational achievement, social integration, and long-term well-being, perpetuating cycles of disadvantage across generations.^11^

Schools represent a unique and potentially transformative platform for delivering mental health interventions to conflict-affected adolescents.^12^ Unlike clinical settings, schools offer several strategic advantages: they provide natural access to large numbers of young people,^13^ reduce stigma associated with mental health service utilization,^14^ integrate seamlessly into existing community infrastructure, and can be implemented by trained teachers or school personnel rather than specialized mental health professionals who are often scarce in conflict-affected regions.^15^ Furthermore, school-based interventions can simultaneously address educational disruption while promoting psychological recovery, recognizing the interconnected nature of academic and mental health outcomes.^16^^,^^17^

Despite increasing recognition of the mental health needs of conflict-affected adolescents in the Middle East, evidence on school-based psychosocial interventions remains fragmented. Prior reviews have focused on refugees more broadly or on programs in high-income settings,^18^ with limited attention to Middle Eastern contexts, although contextual factors are known to influence intervention delivery and effectiveness across settings globally. In Middle Eastern contexts, cultural, religious, and sociopolitical factors, such as gender norms, collectivist values, religious coping, and ongoing conflict, may particularly shape how interventions are implemented and experienced.^19^

This systematic review and meta-analysis evaluate the effectiveness of school-based psychosocial interventions for refugee and conflict-affected adolescents in Middle Eastern countries. By synthesizing evidence across outcomes including PTSD, depression, anxiety, psychosocial functioning, and academic performance, the review aims to identify promising approaches, explore potential moderators, and highlight evidence gaps to inform policy and practice for this vulnerable population.

## Methods

This systematic review and meta-analysis was conducted in accordance with the Preferred Reporting Items for Systematic Reviews and Meta-Analyses 2020 guidelines and the Cochrane handbook.^20^^,^^21^ The review protocol was prospectively registered in the International Prospective Register of Systematic Reviews under registration number CRD420251175178.

### Eligibility criteria

We included quantitative and qualitative studies evaluating school-based psychosocial interventions for refugee or conflict-affected adolescents in Middle Eastern countries. Eligibility criteria were defined using the PICO framework as follows:

Population: Refugee or conflict-affected adolescents aged 10–19 years (World Health Organization (WHO) definition), displaced internally or across borders due to armed conflict, or directly exposed to conflict-related violence in Middle Eastern countries.

Intervention: Structured, non-pharmacological psychosocial programs delivered within school settings to improve psychological, emotional, or behavioral functioning.

Comparator: Waitlist, usual care, no-intervention controls, or alternative school-based activities. Single-arm pre–post studies were also eligible.

Outcomes: Primary outcomes were PTSD and stress-related symptoms. Secondary outcomes included depression, anxiety, emotional and behavioral difficulties, and psychosocial well-being, measured using validated instruments.

Only articles published in English were considered. Exclusion criteria included reviews, editorials, commentaries, studies involving non-refugee populations, community-based (non-school) interventions, and studies conducted outside the Middle East.

### Information sources and search strategy

A comprehensive literature search was conducted in PubMed, Scopus, and Web of Science from database inception to August 2025. The search strategy combined controlled vocabulary and free-text keywords related to refugees, adolescents, psychosocial interventions, school-based programs, and Middle Eastern countries. Reference lists of included studies and relevant reviews were also hand-searched to identify additional relevant articles. The full search strategy for each database is provided in Supplementary Table S1.

### Study selection

All records identified through database searches were imported into EndNote, where duplicates were removed. Two independent reviewers screened titles and abstracts against the eligibility criteria. Full texts of potentially eligible articles were retrieved and assessed for inclusion. Discrepancies were resolved by consensus or by consulting a third reviewer.

### Data extraction

Data from the included studies were independently extracted by two reviewers using a standardized form. Extracted information covered study characteristics (author, year, country, design), participant characteristics (sample size, age, gender, refugee group), and intervention details (type, duration, delivery agent, modality); where applicable, comparator conditions were also noted. Outcomes of interest included PTSD, anxiety, depression, and emotional and behavioral difficulties.

### Risk of bias assessment and certainty of evidence

The Revised Cochrane risk-of-bias tool for randomized trials was used to evaluate RCTs across domains, including randomization, deviations from intended interventions, missing outcome data, outcome measurement, and selection of reported results.^22^ Cohort studies were assessed using the Newcastle–Ottawa Scale (NOS), which evaluates selection, comparability, and outcome domains.^23^ Two reviewers independently assessed risk of bias, with disagreements resolved through discussion.

The certainty of evidence was assessed using the Grading of Recommendations Assessment, Development, and Evaluation approach.^24^ The following domains were evaluated: risk of bias, inconsistency, indirectness, imprecision, and publication bias.

### Data synthesis and statistical analysis

Single-arm analyses (pre–post intervention effects within groups) and double-arm analyses (intervention versus control) were performed using R programming. Effect sizes for continuous outcomes were pooled using either mean raw differences (MRAW) or standardized mean differences (SMD) with 95% confidence interval (CI), depending on the consistency of outcome measures. A random-effects model (DerSimonian and Laird method) was applied due to anticipated heterogeneity. Heterogeneity was assessed using the *I*^2^ statistic and corresponding *P*-value, with *P*-values below .1 indicating substantial heterogeneity. Forest plots were generated to visualize pooled effects. Qualitative findings were synthesized narratively, highlighting themes related to intervention processes and participant experiences.

## Results

**Figure 1:**
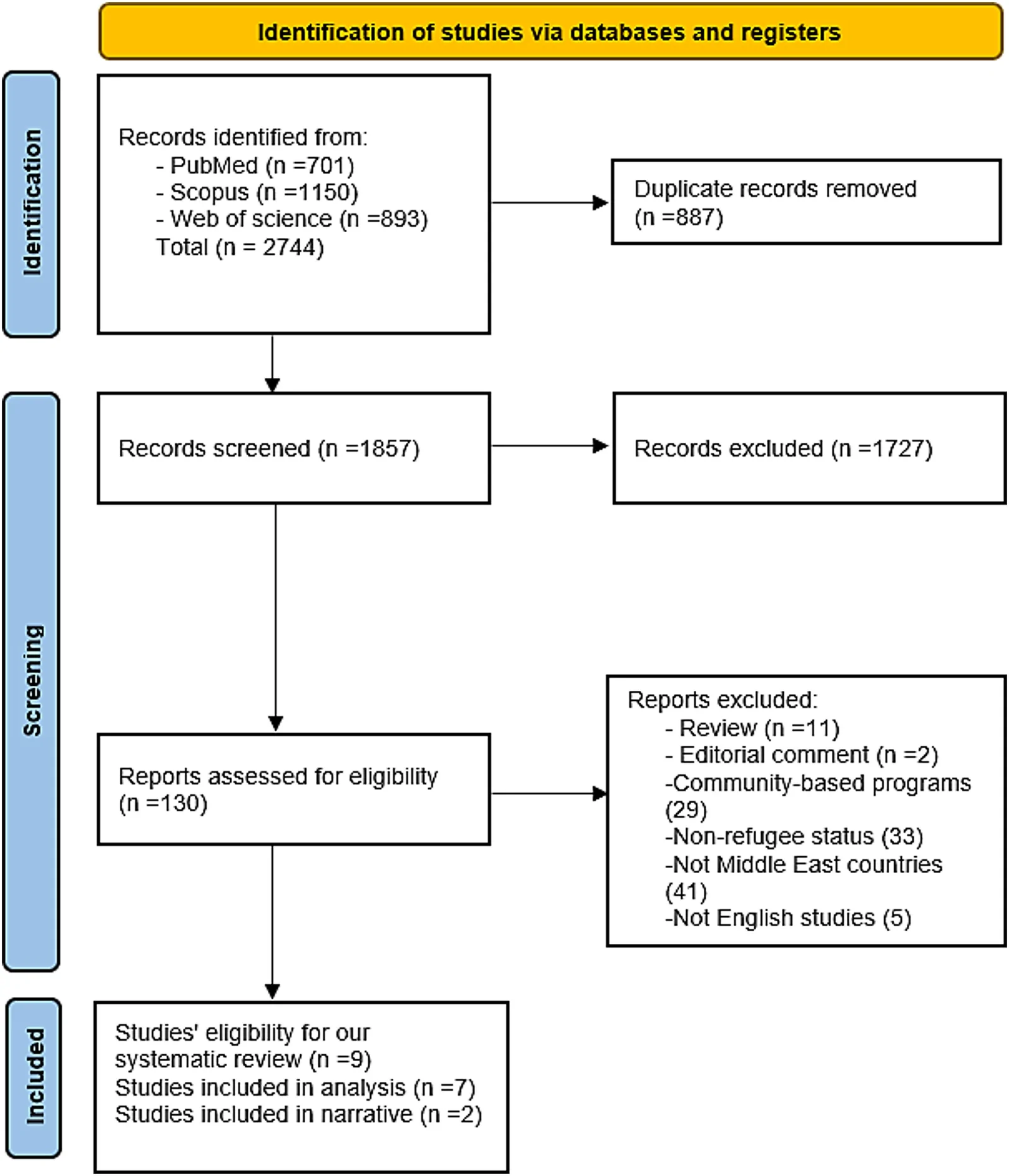
Preferred Reporting Items for Systematic Reviews and Meta-Analyses (PRISMA) flow diagram of study selection.

A total of 2744 records were retrieved from PubMed (701), Scopus (1150), and Web of Science (893). After removing 887 duplicates, 1857 records underwent title and abstract screening. Of these, 130 full-text reports were assessed for eligibility. Exclusions included reviews (11), editorial comments (2), community-based programs (9), non-refugee status (13), studies outside Middle Eastern countries (29), non-refugee status (33), not Middle East countries (41), and non-English articles (5). Ultimately, nine studies met eligibility criteria,^25–33^ with seven included in quantitative analysis^25–31^ and two in narrative synthesis (Fig. 1).^32^^,^^33^

### Baseline and characteristics of the included studies

Sample sizes ranged from 32^27^ to 300 participants,^26^ with mean ages spanning 9–16.65^25^^,^^31^ years. Gender distribution varied, with some studies reporting balanced samples,^30^^,^^32^ while others involved predominantly male participants.^29^^,^^31^ At baseline, interventions addressed PTSD (23.9–56.3), anxiety (12.47–53.3), depression (2.69–14.05), and emotional and behavioral difficulties (15.0–53.0). Interventions were diverse, such as Teaching Recovery Techniques, Cognitive Behavioral Therapy, Writing for Recovery, Better Learning Program level 2, and newer approaches like MemFlex and the Helping Hand mobile application. Delivery agents, including teachers, instructors, and psychologists, also varied, with intervention durations ranging from half a week to 8 weeks. Refugee populations were primarily Syrian,^25^^,^^27^^,^^29^^,^^32^^,^^33^ Palestinian,^26^^,^^30^ and Afghan^28^^,^^31^ (Tables 1 and 2).

**Table 1:** Baseline characteristics of the included studies.

ID	Study groups	Age (years), mean (SD)	Male, *N* (%)	PTSD score, mean (SD)	Anxiety score, mean (SD)	Depression score, mean (SD)	Emotional and behavioral difficulties score mean (SD)
Mirabolfathi *et al*. 2024	MemFlex (*n* = 20)	16.65 (1.42)	6 (30)	NR	NR	NR	53.00 (14.91)
	Waitlist (*n* = 20)	16.60 (1.54)	5 (25)	NR	NR	NR	52.75 (13.73)
Raknes *et al*. 2024	HH app (*n* = 104)	13.38 (1.14)	48 (46.2)	NR	NR	NR	NR
Forsberg and Schultz 2022	BLP-2 (*n* = 200)	11.79 (1.53)	110 (55)	31.34 (11.53)	NR	NR	NR
	Control (*n* = 100)	12.45 (1.86)	40 (40)	30.63 (13.23)	NR	NR	NR
El-Khani *et al*. 2021	TRT + P (*n* = 41)	(9–12)	NR	NR	35.14 (12.11)	13.62 (5.64)	16.75 (5.22)
	TRT (*n* = 38)				34.15 (13.19)	13.32 (5.86)	15.60 (4.53)
	Waitlist (*n* = 40)				34.66 (13.69)	11.34 (6.52)	15.05 (3.63)
Kubitary and Alsaleh 2018	TRPPT (*n* = 41)	14.94 (0.93)	41 (100)	32.7 (10.7)	NR	NR	NR
Sirin *et al*. 2018	Project Hope (*n* = 75)	11.75 (1.23)	NR	NR	NR	2.69 (2.8)	NR
	Wait-list Control (*n* = 72)	11.75 (1.23)	NR	NR	NR	NR	NR
Gormez *et al*. 2017	CBT (*n* = 32)	12.41 (1.68)	12 (37.5)	23.9 (12.80)	53.28 (13.78)	NR	18.77 (4.28)
Kalantari *et al.* 2012	WfR (*n* = 29)	10.77 (3.71)	13 (44.8)	56.3 (11.6)	NR	NR	NR
	Control (*n* = 32)	10.77 (3.71)	16 (50)	49.9 (13.3)	NR	NR	NR
Lange-Nielsen *et al*. 2012	WfR (*n* = 66)	14.48	33 (50)	28.4 (11.7)	12.47 (7.15)	14.05 (6.12)	NR
	WLC (*n* = 58)	14.6	29 (50)	29.4 (11.8)	12.47 (6.02)	13.78 (5.79)	NR

BLP-2 = Better Learning Program level 2; CBT = Cognitive Behavioral Therapy; HH app = Helping Hand mobile application; MemFlex = Memory Specificity Training (Memory Flexibility); NR = Not reported; P = Parenting component; TRPPT = Treatment by Repeating Phrases of Positive Thoughts; TRT = Teaching Recovery Techniques; WfR = Writing for Recovery; WLC = Waitlist Control.

### Outcomes and measures

Primary and secondary mental health outcomes were assessed using validated psychometric instruments across the included studies. PTSD was measured with tools such as the Children’s Revised Impact of Event Scale,^25^^,^^26^^,^^30^ the Child Post-Traumatic Stress Reaction Index,^27^ the Trauma Grief Inventory for Children,^28^ and the Davidson Trauma Scale.^29^

Anxiety outcomes were evaluated using the Screen for Child Anxiety Related Emotional Disorders,^25^ the Revised Children’s Manifest Anxiety Scale,^30^ and the Spence Children’s Anxiety Scale.^27^ Depression was assessed with the Depression Rating Scale^25^^,^^30^ and a hopelessness scale to capture negative expectations about the future.^33^ Emotional and behavioral difficulties were measured using the Strengths and Difficulties Questionnaire^25^^,^^27^ and the Hopkins Symptom Checklist.^31^

### Quality assessment

**Figure 2:**
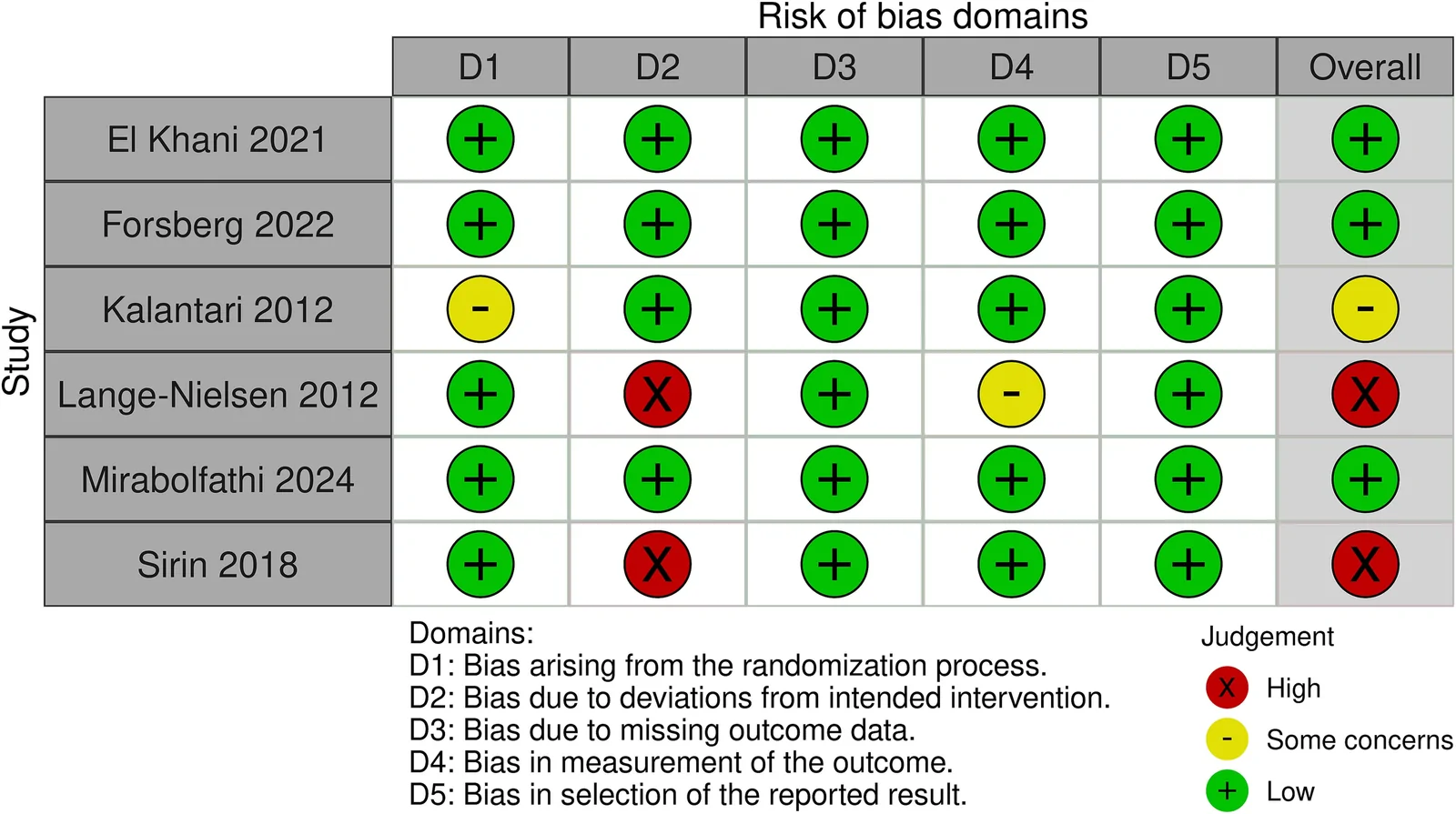
Risk of bias summary for randomized controlled trials, assessed using the Cochrane risk of bias tool.

Regarding the included RCTs, three studies^25^^,^^26^^,^^31^ showed low risk of bias across all domains. Kalantari *et al*. raised some concerns related to randomization.^28^ In contrast, Lange-Nielsen *et al*. and Sirin *et al*. were rated high risk overall, mainly due to deviations from intended interventions and outcome measurement (Fig. 2).^30^^,^^33^

Regarding the included cohort studies, two^27^^,^^29^ were judged as good quality based on the NOS, demonstrating adequate selection, comparability, and outcome assessment. In contrast, Raknes *et al*. were rated as poor quality, mainly due to limitations in outcome assessment and follow-up adequacy (Supplementary Table S2).^32^

### Meta-analysis

#### Single-arm analysis

**Figure 3:**
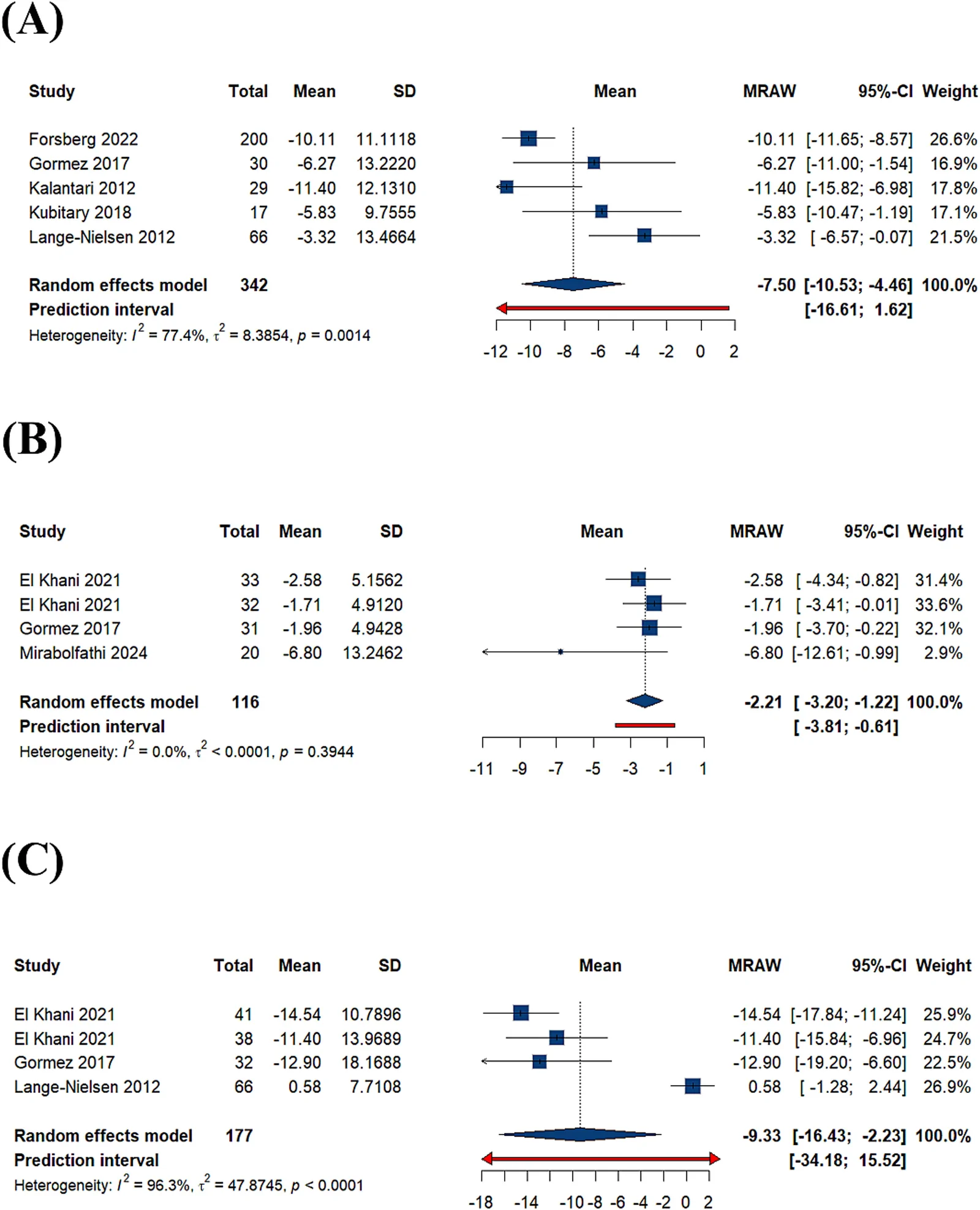
Forest plots of single-arm meta-analyses; (A) reduction in PTSD symptoms following school-based psychosocial interventions; (B) reduction in emotional and behavioral difficulties; (C) reduction in anxiety symptoms.

The meta-analysis of PTSD outcomes included five studies with 342 participants. It showed a significant reduction in PTSD symptoms following school-based interventions (MRAW = −7.50, 95% CI: −10.53 to −4.46), though with high heterogeneity (*I*^2^ = 77.4%, *P* = .0014). For emotional and behavioral difficulties (Fig. 2), three studies with 116 participants demonstrated a modest but consistent improvement through school-based programs (MRAW = −2.21, 95% CI: −3.20 to −1.22; *I*^2^ = 0.0%, *P* = .3944). Anxiety outcomes were reported in three studies with 177 participants, showing a significant reduction with school-based interventions (MRAW = −9.33, 95% CI: −16.43 to −2.23), but with substantial heterogeneity (*I*^2^ = 96.3%, *P* < .0001) (Fig. 3A–C).

**Figure 4:**
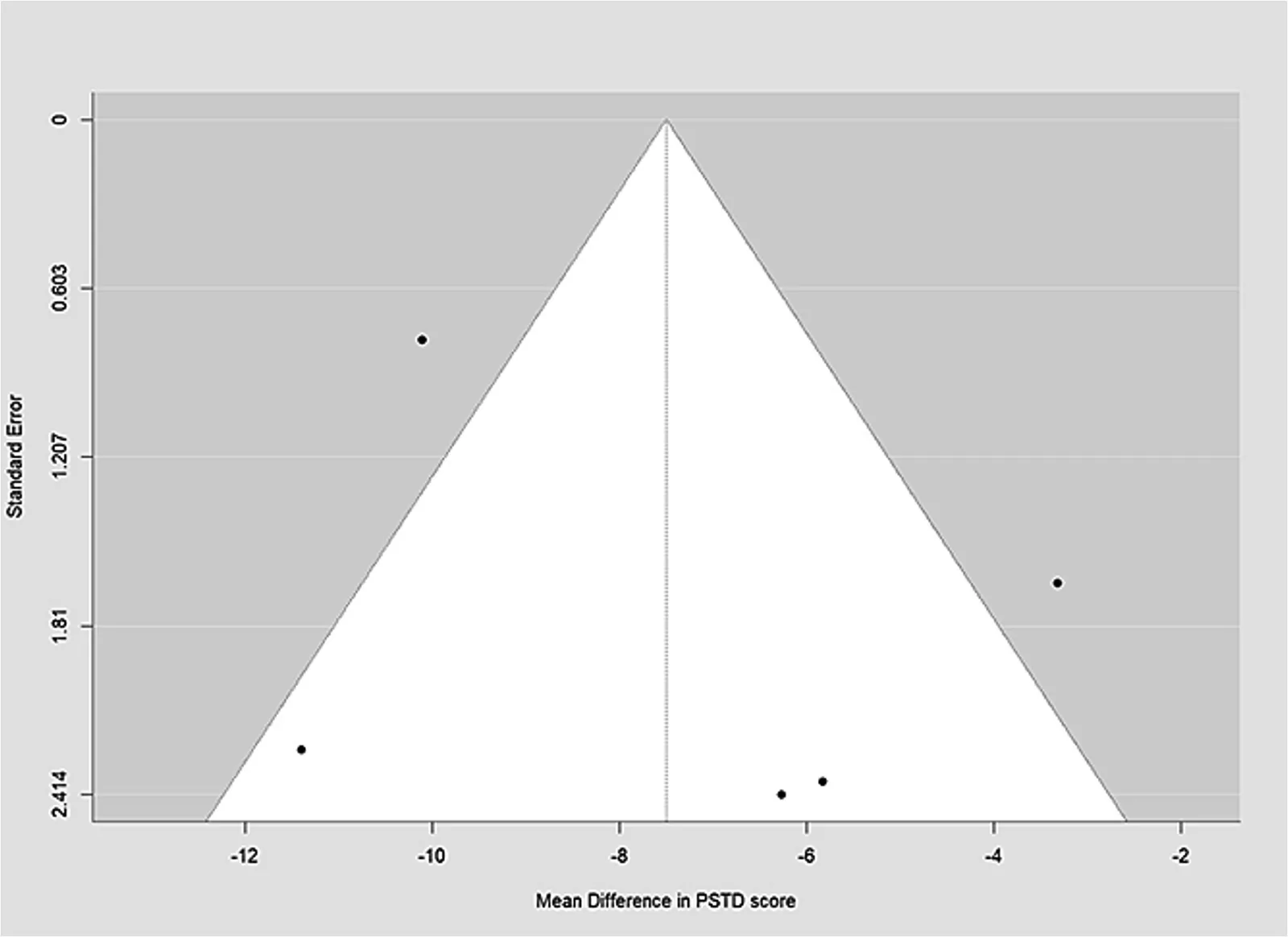
Funnel plot of PTSD outcome.

The funnel plot of PTSD outcomes appears visually symmetrical around the pooled effect size, with studies distributed on both sides of the mean difference and no clear evidence of small-study effects (Fig. 4).

**Table 2:** Summary of the included studies.

ID	Study design	Country	Refugee/status	Sample size	Intervention	Control	Delivery agents	Duration and setting	Outcomes	Measurement tools	Main findings
**Mirabolfathi 2024**	RCT	Iran	Afghan	40	MemFlex: A structured memory-based psychological program targeting autobiographical memory processes, which trains participants to improve retrieval of specific, detailed personal memories and to reduce negative memory bias by enhancing access to positive, self-affirming memories.	Waitlist	Clinical psychologist	4 weekly group-based face-to-face sessions +4 self-guided workbook sessions	Memory flexibility and specificity Future thinking Self-cognitions General emotional distress	Autobiographical Memory Task—Alternating Instructions (AMT-AI) Future Thinking Task Twenty Statement Test The 25-item Hopkins Symptom Checklist	Improved emotional distress Improvement in memory biases Enhanced positive future thinking Limited impact on trauma-related self-cognitions
**Raknes 2024**	Prospective cohort	Lebanon	Syrian	104	Happy Helping Hand (HH): A digital social and emotional learning (SEL) mobile application based on CBT principles. The app uses role-playing game scenarios to teach emotional problem-solving skills (problem identification, emotional awareness, red/green thoughts, coping strategies, help-seeking).	N/A	Teachers (MAPS-trained)	5 weeks; 10 sessions (2 sessions/week); school-based blended learning (15 min gameplay +60 min group discussion per session)	Well-being Emotional and social problem-solving skills	WHO-5 Well-Being Index (Arabic version) Semi-structured focus groups	Significant increase in well-being (WHO-5) Improved social and emotional skills Increased self-confidence, decision-making, and help-seeking behavior
**Forsberg 2022**	RCT	Palestine	Palestinian	300	Better Learning Program 2 (BLP-2): A school-based, teacher-led psychosocial program combining CBT-based stress management (psychoeducation, relaxation, coping skills, social support, parent involvement) with educational components (study skills, self-efficacy, academic support).	Waitlist Control (WLC)	Teachers (trained in BLP-2)	5 weeks; 5 structured sessions (45 min each); small groups; school-based	Well-being; Self-regulation; Self-efficacy; Executive function/study skills; Self-perceived academic functioning Stress-related symptoms Academic performance (Arabic & Math grades)	Author-developed 17-item self-report tool Children’s Impact of Event Scale (CRIES-13) School grades (Arabic & Math)	Improvement and medium to large effect sizes in all the measured domains
**El Khani 2021**	RCT	Lebanon	Syrian	119	Teaching Recovery Techniques (TRT) + Parenting: Trauma-focused CBT-based child group intervention enhanced with 3 additional caregiver parenting sessions focusing on positive parenting skills, emotional regulation, behaviour management, and caregiver wellbeing.	Waitlist Control (WLC)	Trained teachers/ facilitators	5-week program; weekly group sessions (child and caregiver sessions delivered separately in school setting)	Child PTSD symptoms Child depressive symptoms Child anxiety disorders Parenting skills and confidence Caregiver Post-Traumatic Stress symptoms Caregiver depression, anxiety, and stress	Children’s Impact of Event Scale (CRIES-13) The Depression Self-Rating Scale for Children (DSRS) The Screen for Childhood Anxiety-Related Disorders (SCARED) The Parenting Scale (PS) The Impact of Events Scale Revised (IES-R) The Depression–Anxiety–Stress Scale (DASS)	Great reductions in child PTSD (intrusion, avoidance, arousal), depression, and anxiety. Improved parenting skills and parenting confidence. Significant reductions in caregiver depression, anxiety, stress, and PTSD symptoms.
**Kubitary 2018**	Prospective cohort	Syria	Syrian	41	Treatment by Repeating Phrases of Positive Thoughts (TRPPT): Cognitive and positive psychotherapy intervention involving daily repetition of positive phrases, cognitive restructuring, thought monitoring, and positive self-talk training.	N/A	Psychologist	5 weeks; daily practice (3–5 min morning & evening); school-based training with home practice	PTSD symptoms Sleep disorders War experiences	Davidson Trauma Scale (DTS) Sleep Disorders Scale (SDS) Kubitary-AlsalehWar Experiences Scale (KAWES)	Significant reduction in PTSD symptoms, sleep disorders, and war experiences.
**Sirin 2018**	RCT	Turkey	Syrian	147	Project Hope: A digital game-based educational intervention combining Turkish language training (Cerego), executive function training (Alien Game), coding instruction (Code.org), and Minecraft-based activities to enhance hopefulness.	Waitlist Control (WLC)	Trained instructors	4 weeks; 5 days/week; 2 hours/day (total 40 hours); school-based computer lab sessions	Turkish language skills Executive function (shifting) Hopelessness	Turkish vocabulary pre/post tests The dimensional change card sort task (DCCS) Beck’s hopelessness scale	Significant improvements in Turkish language, executive function, and reduced hopelessness
**Gormez 2017**	Prospective cohort	Turkey	Syrian	32	CBT program: Manualized 8-session cognitive-behavioral psychological support intervention targeting trauma-related symptoms, anxiety, emotional regulation, coping skills, relaxation techniques, cognitive restructuring, trauma narrative processing, and problem-solving skills.	N/A	Trained teachers	8 weekly sessions (70–90 min each); small groups; school-based	PTSD symptoms Anxiety Emotional & behavioral problems	Child Post-Traumatic Stress Reaction Index (CPTS-RI) Spence children’s anxiety scale (SCAS) Strengths and difficulties questionnaires (SDQ)	Significant reduction in anxiety and PTSD total scores Emotional problems improved in SDQ No significant change in conduct, hyperactivity, and peer-relationships related problem areas
**Kalantari 2012**	RCT	Iran	Afghan	61	Writing for Recovery: Structured group expressive writing intervention progressing from unstructured emotional writing about loss/trauma to structured reflection, advice-giving, and future-oriented meaning-making exercises.	N/A	Research team facilitators	3 consecutive days; 2 sessions/day (15 min each); group-based school setting	Traumatic grief symptoms	Traumatic Grief Inventory for Children (TGIC)	Significant reduction in traumatic grief symptoms
**Lange-Nielsen 2012**	RCT	Palestine	Palestinian	124	Writing for Recovery: Manual-based structured expressive writing intervention involving trauma-focused narrative writing, sensory processing of traumatic memories, cognitive reframing, and meaning-making exercises.	Waitlist Control (WLC)	Professional psychologists	3 consecutive days; 2 sessions/day (15 min each); group-based school setting	PTSD symptoms Depression Trauma exposure Anxiety	Children’s Impact of Event Scale (CRIES-13) The Depression Self-Rating Scale for Children (DSRS) Gaza Traumatic Event Checklist (GTECL) Revised Children’s Manifest Anxiety Scale (RCMAS)	No significant intervention effect Significant short-term increase in depression; reduced at follow-up with no significant difference from baseline

CBT = Cognitive behavioral therapy, MAPS = Multi-aid programs, N/A = Not applicable, NR = Not reported.

**Figure 5:**
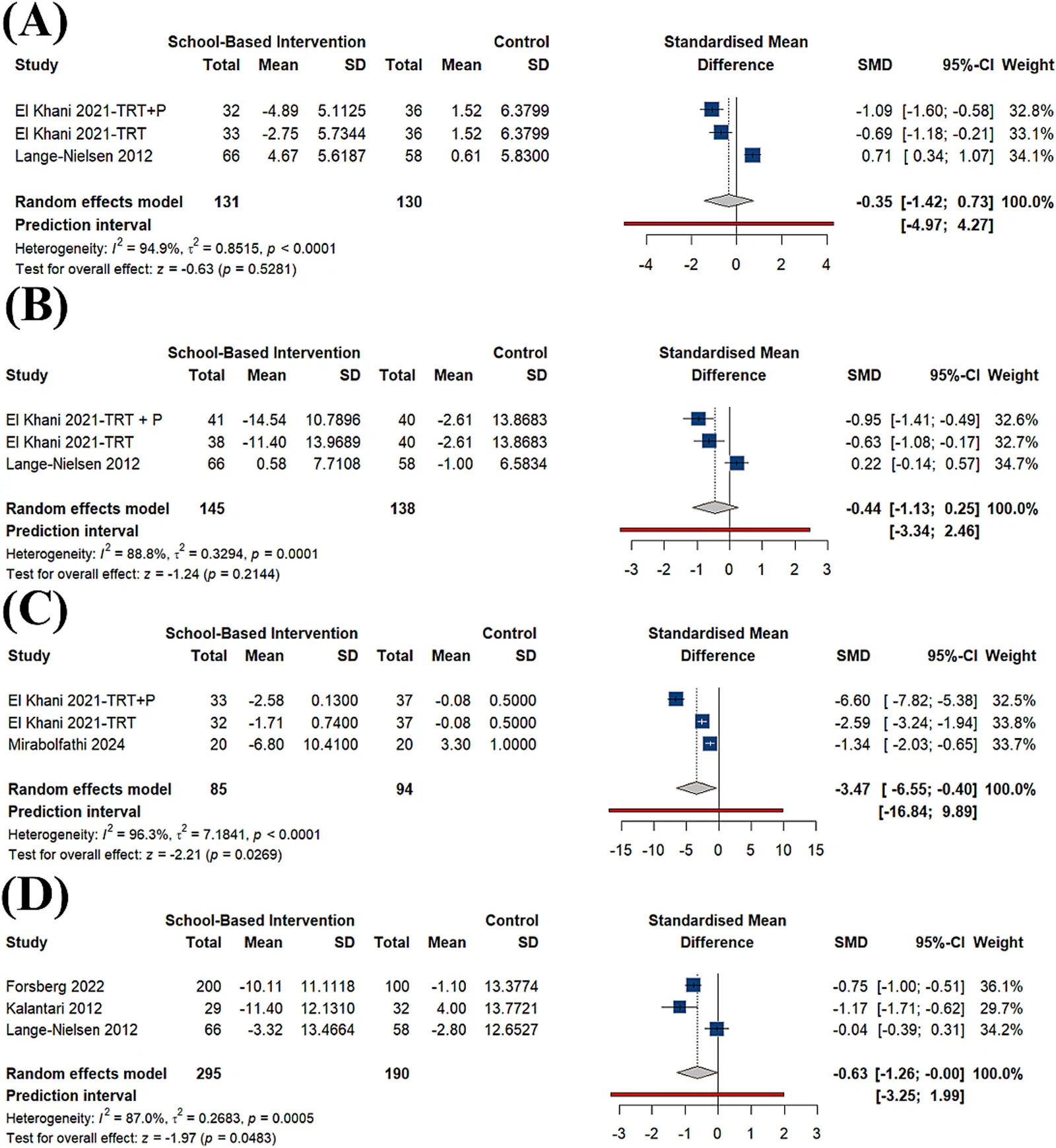
Forest plots of double-arm meta-analyses comparing school-based psychosocial interventions to control conditions; (A) effects on depression outcomes; (B) effects on anxiety outcomes; (C) effects on emotional and behavioral difficulties; (D) effects on stress/PTSD outcomes.

#### Double-arm analysis

For depression, two studies with 161 participants found no significant difference between school-based programs and control groups (SMD = −0.35, 95% CI: −1.42 to 0.73, *P* = .53), with very high heterogeneity (*I*^2^ = 94.9%, *P* < .0001). Similarly, anxiety outcomes from two studies (145 intervention, 138 control participants) showed no significant reduction (SMD = −0.44, 95% CI: −1.13 to 0.25, *P* = .21), again with high heterogeneity (*I*^2^ = 88.8%, *P* = .0001). In contrast, two studies assessing emotional and behavioral difficulties reported a significant improvement in the intervention groups compared to controls (SMD = −3.47, 95% CI: −6.55 to −0.40, *P* = .027), though heterogeneity remained high (*I*^2^ = 96.3%, *P* < .0001). Finally, three studies evaluating stress and PTSD outcomes showed a significant reduction among participants receiving school-based interventions relative to control groups (SMD = −0.63, 95% CI: −1.26 to −0.01, *P* = .048), with substantial heterogeneity (*I*^2^ = 87.0%, *P* = .0005) (Fig. 5A–D).

#### Regression analysis

Egger’s regression test for the PTSD outcome, using a mixed-effects meta-regression model, showed no significant evidence of funnel plot asymmetry (*z* = 0.4636, *P* = .6430). The non-significant *P*-value suggests that small-study effects or publication bias for PTSD are unlikely. The limit estimate as the standard error approaches zero was −9.67 (95% CI: −19.57 to 0.23), indicating that the adjusted pooled PTSD effect remains similar in magnitude, although the CI crosses zero, reflecting imprecision.

### Qualitative synthesis

Raknes *et al*. evaluated a 5-week, 10-session digital social and emotional learning program (Happy Helping Hand app) delivered to displaced Syrian adolescents in Lebanon.^32^ The intervention focused on emotional awareness, cognitive coping strategies, problem-solving skills, and self-management through interactive digital modules. Participants demonstrated a statistically significant increase in overall well-being. Qualitative findings indicated high acceptability, with adolescents reporting improved emotional regulation, greater self-confidence, enhanced problem-solving abilities, and increased self-awareness. The digital format was perceived as engaging and accessible, supporting feasibility in resource-constrained and displacement settings.

Similarly, Sirin *et al*. assessed Project Hope, a 4-week, 40-hour online game-based educational intervention for Syrian refugee children in Turkey.^33^ The program combined language acquisition, executive function training, and psychosocial skill-building within a structured digital learning environment. Beyond reductions in hopelessness, participants reported improved motivation, future orientation, and engagement with learning tasks. The structured, instructor-supported format enhanced adherence and acceptability, suggesting feasibility within school-based contexts serving refugee populations.

Across studies, common intervention elements included trauma-focused components (e.g. exposure or trauma narration), cognitive restructuring, emotional regulation strategies, structured group discussions, and skill-building exercises. Writing-based interventions (e.g. Writing for Recovery) emphasized narrative processing of traumatic experiences, while cognitive-behavioral approaches incorporated coping skills and behavioral activation. Teacher- or instructor-led delivery models were frequently used, supporting scalability in low-resource settings.

### Certainty of evidence

**Figure 6:**
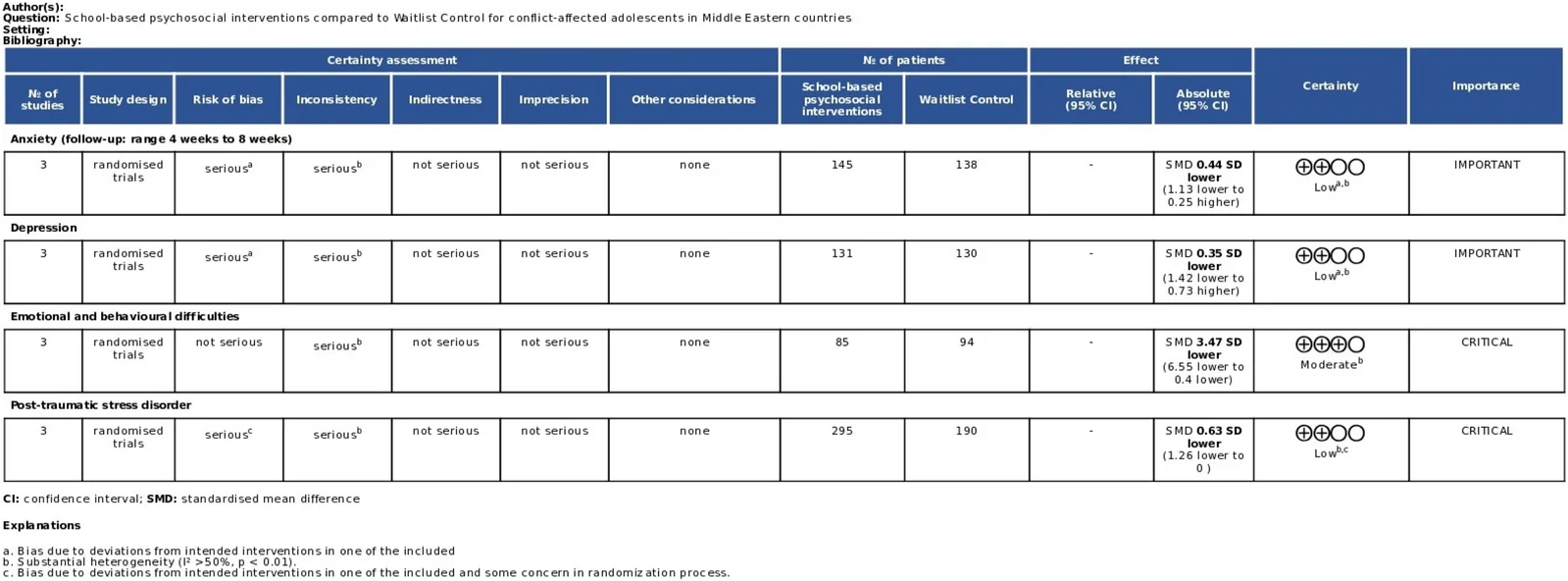
The Grading of Recommendations Assessment, Development, and Evaluation (GRADE) assessment for the study outcomes.

Across three RCTs per outcome, evidence was rated as low certainty for anxiety, depression, and PTSD, and moderate certainty for emotional and behavioral difficulties. Downgrading was primarily due to serious risk of bias and substantial heterogeneity (*I*^2^ >50%). Interventions showed reductions in anxiety (SMD 0.44 lower), depression (SMD 0.35 lower), emotional and behavioral difficulties (SMD 3.47 lower), and PTSD symptoms (SMD 0.63 lower) compared with waitlist control. Anxiety and depression were judged important outcomes, while emotional/behavioral difficulties and PTSD were considered critical (Fig. 6).

## Discussion

### Main finding of this study

The present systematic review and meta-analysis demonstrate that school-based psychosocial interventions yield significant improvements in mental health outcomes among conflict-affected adolescents in Middle Eastern countries. However, effect sizes and consistency vary across symptom domains. Single-arm pre–post analyses revealed substantial reductions in PTSD symptoms, anxiety, and emotional and behavioral difficulties. These within-group effect sizes indicate clinically meaningful symptom reduction following intervention delivery, consistent with established minimal clinically significant differences for trauma-related disorders in adolescent populations.^34^

However, double-arm analyses comparing intervention and control groups presented a more differentiated pattern of effects. Significant treatment effects were observed for PTSD and stress symptoms, emotional and behavioral difficulties, whereas no significant differences emerged for depression or when compared with control conditions. This divergence between single-arm and double-arm results warrants careful interpretation and suggests several explanatory mechanisms.

First, the observed improvements in control groups may reflect natural recovery patterns associated with environmental stabilization, a phenomenon well-documented in trauma literature.^35^ Adolescents in control conditions nonetheless benefited from school attendance, which provides structure, social support, and respite from adverse living conditions—factors known to facilitate spontaneous recovery from acute stress reactions.^36^ Second, the chronicity and ongoing nature of stressors faced by displaced populations may attenuate intervention effects for internalizing symptoms such as depression and anxiety, which are particularly sensitive to current life circumstances rather than past traumatic events alone.^37^ Time-limited school interventions, regardless of their theoretical grounding or delivery quality, may prove insufficient to counteract persistent daily stressors, including poverty, legal uncertainty, family separation, and discrimination.

Third, the substantial heterogeneity observed across studies reflects genuine variation in intervention characteristics, population demographics, conflict exposure profiles, and contextual factors. Syrian refugees in Lebanon experience markedly different post-migration stressors compared to Palestinian adolescents in the West Bank or Afghan refugees in Iran, potentially moderating treatment response. This heterogeneity, while statistically challenging, accurately represents the complexity of delivering mental health interventions across diverse humanitarian contexts.^38^ The most consistent benefits were seen for trauma symptoms and emotional regulation, which were the main targets of the interventions.^39^

Intervention safety is an important consideration in school-based psychosocial programs. Although trauma-focused approaches may carry a risk of temporary distress if inadequately supported, none of the included studies reported serious adverse events. Evidence suggests such interventions are generally safe when delivered with appropriate training and supervision. However, reporting of adverse events and safety monitoring were limited and should be strengthened in future research.^40^

### What is already known on this topic

Our findings are consistent with prior systematic reviews of psychosocial interventions for refugee youth, particularly regarding trauma outcomes. Tyrer and Fazel reported small-to-moderate PTSD symptom reductions,^41^ while Sullivan and Simonson found significant effects for trauma patterns mirrored in our results.^42^ Unlike these reviews, which mainly drew from high-income resettlement settings, our analysis focuses on Middle Eastern host countries (Lebanon, Jordan, Turkey, and Palestinian territories), where interventions operate in under-resourced systems serving large, displaced populations. The persistence of treatment effects in these contexts suggests that evidence-based approaches remain effective in humanitarian settings.^43^ However, we found few high-quality trials, with most studies limited by methodological weaknesses. This reflects the challenges of conducting rigorous research in crisis settings, including ethical concerns, population mobility, and strained infrastructures.^38^

### What this study adds

The findings from this review have important implications for practice, implementation, and policy in humanitarian settings. First, the results support integrating mental health programming within education systems serving conflict-affected populations, rather than relying solely on separate referral pathways. School-based delivery reduces barriers such as transport, cost, and stigma.^44^ Investing in teacher training and school-based infrastructure appears cost-effective and scalable, particularly as several interventions were delivered by trained teachers or facilitators rather than mental health professionals, consistent with task-sharing models such as WHO mental health gap action programme (mhGAP),^45^ and demonstrated outcomes comparable to psychologist-led programs in resource-constrained settings.

Second, the differential effects across symptom domains suggest interventions should be matched to primary needs. Programs emphasizing trauma processing and emotional regulation show the most consistent benefits and should be prioritized for adolescents with acute post-traumatic stress. Depression and anxiety, by contrast, may require longer interventions, sustained support, and approaches addressing material and social determinants.^37^ Brief school-based programs are best seen as stabilization, not comprehensive treatment.

Moreover, cultural, religious, and sociopolitical factors in Middle Eastern contexts likely influence intervention delivery and responsiveness. Collectivist values, family cohesion, religious coping, and gender norms may shape participation and emotional expression, while ongoing insecurity can affect engagement and sustainability.^46^^,^^47^ Although rarely examined explicitly, these factors should be considered when interpreting findings and planning culturally responsive adaptations.

Finally, the substantial heterogeneity across studies highlights the need for culturally adapted, contextually responsive programming. Maintaining fidelity to core therapeutic elements must be balanced with flexibility to reflect cultural values, religious practices, gender norms, and local views of mental health.^48^ Implementation frameworks stressing adaptation, stakeholder engagement, and quality improvement are critical for effective delivery in Middle Eastern contexts.

### Methodological strengths and limitations

This review has several strengths. By including both single- and double-arm meta-analyses, we were able to evaluate both pre- and post-changes and intervention effects relative to controls. Furthermore, our exclusive focus on Middle Eastern contexts enhances the direct relevance of findings to a region bearing the world’s highest displacement burden.

Limitations should be noted. Only nine studies were eligible, limiting statistical power and preventing subgroup analyses. High heterogeneity reduced precision and suggested intervention effects may vary by context. Study quality was mixed, with several at high risk of bias and many relying on uncontrolled pre–post designs. Publication bias cannot be excluded, and restricting to English-language reports may have excluded relevant regional studies. Finally, the lack of long-term follow-up prevents conclusions about the durability of effects.

## Conclusions and recommendations for future research

School-based psychosocial interventions show strong potential for reducing trauma symptoms and improving emotional-behavioral functioning among conflict-affected adolescents in the Middle East. Meta-analyses indicate consistent benefits for PTSD and emotional regulation, with mixed effects for depression and anxiety. However, the substantial heterogeneity observed across several analyses limits the certainty and generalizability of these pooled estimates. Effect sizes should therefore be interpreted cautiously, as variability in intervention models, study quality, population characteristics, and contextual factors may influence outcomes. These findings support scaling up evidence-based school programs as part of humanitarian response, while recognizing they alone cannot meet displaced populations’ complex mental health needs.

Future research should focus on adequately powered randomized trials with active controls, implementation factors (training, supervision, cultural adaptation, sustainability), and moderators/mediators of treatment response. Longitudinal studies are needed to test the durability of benefits, and economic evaluations will inform cost-effectiveness in resource-limited contexts.

For practice, the evidence supports integrating culturally adapted, trauma-focused programs into education systems, delivered by trained school staff under supervision. Such integration should be accompanied by ongoing monitoring and rigorous evaluation to better understand which components are most effective under specific contextual conditions. These should form part of broader psychosocial strategies that also address material needs, family support, social integration, and protection concerns shaping adolescent mental health in displacement.

## Supplementary Material

Supplementary_materials_fdag057

## Data Availability

The data that support the findings of this study are available from the corresponding author upon reasonable request.

## Notes

**Amani N. Alansari**, Consultant Pediatric & Trauma Surgeon MD

**Amani Salim**, PHD Public Health Assistant Professor

**Marwa Messaoud**, Associated Professor MD

**Mohammed A. Mahmoud**, Medical Student

**Hanan Youssif**, Consultant Pediatric Surgeon MD
