# Supplementary material for: School-based psychosocial interventions for refugee and conflict-affected adolescents in Middle Eastern countries: a systematic review and meta-analysis
Source: J Public Health (Oxf). 2026 Jul 29;48(3):724–38. doi: 10.1093/pubmed/fdag057 (PMC13531710; doi:10.1093/pubmed/fdag057)
Supplement: Supplementary file 1 [file supplementary_materials_fdag057.zip › Supplementary_materials_fdag057/Supplementary_tables.docx]

**Table S1:** Search strategy for each database.

| **Database** | **Search Query (Field-Specific Syntax)** | **Results** |
| --- | --- | --- |
| **PubMed** | ("refugee"[Title/Abstract] OR "refugees"[Title/Abstract] OR "asylum seeker"[Title/Abstract] OR "asylum seekers"[Title/Abstract] OR "displaced person"[Title/Abstract] OR "displaced persons"[Title/Abstract] OR "immigrant"[Title/Abstract] OR "migrant"[Title/Abstract]) AND ("adolescent*"[Title/Abstract] OR "youth"[Title/Abstract] OR "child*"[Title/Abstract] OR "student*"[Title/Abstract] OR "young"[Title/Abstract] OR "pediatric*"[Title/Abstract] OR "minor*"[Title/Abstract] OR "pupil*"[Title/Abstract] OR "undergraduate*"[Title/Abstract] OR "learner*"[Title/Abstract] OR "trainee*"[Title/Abstract]) AND ("mental health"[Title/Abstract] OR "psychological"[Title/Abstract] OR "psychiatric"[Title/Abstract] OR "emotional"[Title/Abstract] OR "cognitive"[Title/Abstract] OR "behavioral"[Title/Abstract] OR "well-being"[Title/Abstract] OR "distress"[Title/Abstract] OR "depression"[Title/Abstract] OR "anxiety"[Title/Abstract] OR "PTSD"[Title/Abstract] OR "trauma"[Title/Abstract] OR "stress"[Title/Abstract] OR "psychosocial"[Title/Abstract] OR "social support"[Title/Abstract] OR "peer support"[Title/Abstract] OR "social functioning"[Title/Abstract] OR "social connectedness"[Title/Abstract] OR "interpersonal relationship*"[Title/Abstract] OR "family functioning"[Title/Abstract] OR "social isolation"[Title/Abstract] OR "community support"[Title/Abstract]) AND ("Middle East"[Title/Abstract] OR "Saudi Arabia"[Title/Abstract] OR "Iran"[Title/Abstract] OR "Iraq"[Title/Abstract] OR "Turkey"[Title/Abstract] OR "Israel"[Title/Abstract] OR "UAE"[Title/Abstract] OR "Jordan"[Title/Abstract] OR "Lebanon"[Title/Abstract] OR "Kuwait"[Title/Abstract] OR "Oman"[Title/Abstract] OR "Qatar"[Title/Abstract] OR "Bahrain"[Title/Abstract] OR "Syria"[Title/Abstract] OR "Yemen"[Title/Abstract] OR "Palestine"[Title/Abstract] OR "Egypt"[Title/Abstract] OR "Gaza"[Title/Abstract]) | **701** |
| **Scopus** | ABS ( ( "refugee" OR "refugees" OR "asylum seeker" OR "asylum seekers" OR "displaced person" OR "displaced persons" OR "immigrant" OR "migrant" ) AND ( "adolescent*" OR "youth" OR "child*" OR "student*" OR "young" OR "pediatric*" OR "minor*" OR "pupil*" OR "undergraduate*" OR "learner*" OR "trainee*" ) AND ( "mental health" OR "psychological" OR "psychiatric" OR "emotional" OR "cognitive" OR "behavioral" OR "well-being" OR "distress" OR "depression" OR "anxiety" OR "PTSD" OR "trauma" OR "stress" OR "psychosocial" OR "social support" OR "peer support" OR "social functioning" OR "social connectedness" OR "interpersonal relationship*" OR "family functioning" OR "social isolation" OR "community support" ) AND ( "Middle East" OR "Saudi Arabia" OR "Iran" OR "Iraq" OR "Turkey" OR "Israel" OR "UAE" OR "Jordan" OR "Lebanon" OR "Kuwait" OR "Oman" OR "Qatar" OR "Bahrain" OR "Syria" OR "Yemen" OR "Palestine" OR "Egypt" OR "Gaza" ) ) | **1150** |
| **Web of Science** | AB=(("refugee" OR "refugees" OR "asylum seeker" OR "asylum seekers" OR "displaced person" OR "displaced persons" OR "immigrant" OR "migrant") AND ("adolescent*" OR "youth" OR "child*" OR "student*" OR "young" OR "pediatric*" OR "minor*" OR "pupil*" OR "undergraduate*" OR "learner*" OR "trainee*") AND ("mental health" OR "psychological" OR "psychiatric" OR "emotional" OR "cognitive" OR "behavioral" OR "well-being" OR "distress" OR "depression" OR "anxiety" OR "PTSD" OR "trauma" OR "stress" OR "psychosocial" OR "social support" OR "peer support" OR "social functioning" OR "social connectedness" OR "interpersonal relationship*" OR "family functioning" OR "social isolation" OR "community support") AND ("Middle East" OR "Saudi Arabia" OR "Iran" OR "Iraq" OR "Turkey" OR "Israel" OR "UAE" OR "Jordan" OR "Lebanon" OR "Kuwait" OR "Oman" OR "Qatar" OR "Bahrain" OR "Syria" OR "Yemen" OR "Palestine" OR "Egypt" OR "Gaza")) | **893** |

**Table S2:** Quality assessment of Cohort studies with the NOS tool**.**

| **Study ID** | **Selection** | | | | **Comparability** | **Outcome** | | | **Overall judgment** |
| --- | --- | --- | --- | --- | --- | --- | --- | --- | --- |
|  | D1 | D2 | D3 | D4 |  | D5 | D6 | D7 |  |
| Gromez 2017 | * | * | * | * | ** | * |  | * | Good |
| Kubitary 2018 | * | * | * | * | ** | * |  | * | Good |
| Raknes 2024 | * | * | * | * | * | * |  |  | Poor |

D1: Is the case definition adequate/Representative of the exposed cohort?

D2: Representative of the cases/Selection of the non-exposed cohort.

D3: Selection of Controls/Ascertainment of exposure.

D4: Definition of Controls/ Demonstration that outcome of interest was not present at the start of the study.

D5: Ascertainment of exposure/ Assessment of outcome.

D6: Same method of ascertainment for cases and controls/ Was follow-up long enough for outcomes to occur?

D7: Non-response rate/ Adequacy of follow-up of cohorts.
